# Prevalence and management of severe asthma in primary care: an observational cohort study in Sweden (PACEHR)

**DOI:** 10.1186/s12931-018-0719-x

**Published:** 2018-01-18

**Authors:** Kjell Larsson, Björn Ställberg, Karin Lisspers, Gunilla Telg, Gunnar Johansson, Marcus Thuresson, Christer Janson

**Affiliations:** 1 Work Environment Toxicology, The National Institute of Environmental Medicine, Karolinska Institutet, Stockholm, Sweden; 2 Department of Public Health and Caring Sciences, Family Medicine and Preventive Medicine, Uppsala University, Uppsala, Sweden; 3 AstraZeneca Nordic-Baltic, Södertälje, Sweden; 4 Statisticon AB, Uppsala, Sweden; 5 Department of Medical Sciences, Respiratory, Allergy and Sleep Research, Uppsala University, Uppsala, Sweden

**Keywords:** Severe asthma, Asthma prevalence, Uncontrolled asthma

## Abstract

**Background:**

Severe and uncontrolled asthma is associated with increased risk of exacerbations and death. A substantial proportion of asthma patients have poor asthma control, and a concurrent COPD diagnosis often increases disease burden. The objective of the study was to describe the prevalence and managemant of severe asthma in a Swedish asthma popuöation.

**Methods:**

In this observational cohort study, primary care medical records data (2006–2013) from 36 primary health care centers were linked to data from national mandatory Swedish health registries. The studied population (>18 years) had a record of drug collection for obstructive pulmonary disease (ATC code R03) during 2011–2012, and a physician diagnosed asthma (ICD-10 code J45-J46) prior to drug collection. Severe asthma was classified as collection of high dose inhaled steroid (> 800 budesonide or equivalent per day) and leukotriene receptor antagonist and/or long-acting beta-agonist. Poor asthma control was defined as either collection of ≥600 doses of short-acting beta-agonists, and/or ≥1 exacerbation(s) during the year post index date.

**Results:**

A total of 18,724 asthma patients (mean 49 years, 62.8% women) were included, of whom 17,934 (95.8%) had mild to moderate and 790 (4.2%) had severe asthma. Exacerbations were more prevalent in severe asthma (2.59 [2.41–2.79], Relative Risk [95% confidence interval]; *p* < 0.001). Poor asthma control was observed for 28.2% of the patients with mild to moderate asthma and for more than half (53.6%) of the patients with severe asthma (<0.001). Prior to index, one in five severe asthma patients had had a contact with secondary care and one third with primary care. A concurrent COPD diagnosis increased disease burden.

**Conclusion:**

Severe asthma was found in 4.2% of asthma patients in Sweden, more than half of them had poor asthma control, and most patients had no regular health care contacts.

## Background

Asthma treatment has improved during the last 3 decades and mortality because of to asthma has decreased [1]. Notwithstanding, many asthma patients lack full asthma control as defined by international guidelines [2]. In a survey of nearly 3500 patients with asthma, three of four patients inhaled short-acting beta-agonists (SABA) on a daily basis and half of the patients had uncontrolled asthma [3]. In another study almost half of the asthma patients experienced day time symptoms, one third had sleep disturbances, and one of four reported unscheduled emergency/health care visits [4]. Still, half of the patients who reported severe persistent symptoms of asthma considered their asthma to have been well or completely controlled indicating that individuals with asthma becomes accustomed to their symptoms [4].

Severe asthma is associated with high risk of severe exacerbations and death [5], and represents a high proportion of the total costs for asthma care [6, 7]. There is no uniform definition of severe asthma, but according to ERS/ATS and GINA guidelines severe asthma is defined as asthma that requires treatment with high doses of inhaled corticosteroids plus a second controller, and/or systemic corticosteroids, to prevent it from becoming uncontrolled, or remains uncontrolled despite this therapy [2, 8]. Swedish recommendations on asthma management are similar to the GINA guidelines [2], in which it is recommended that severe asthma should be managed by respiratory/allergy specialists [2, 5, 9]. Most patients with asthma have a mild or moderate disease and data on the prevalence of severe asthma is sparse but reports indicate between 5 and 10% of the asthmatic populations to have had severe disease [8, 10]. In a recent observational study, 8.1% were reported to have severe asthma [11]. In that study, severe asthma was defined as a need for a high dosage of inhaled steroids added to a second controller (long-acting beta-agonists (LABA), leukotriene modifiers (LTRA), theophylline or treatment with omalizumab [11]).

Uncontrolled asthma is not always synonymous with severe disease. Poor control may depend on refractoriness to treatment, but may also be caused by other factors such as poor treatment adherence, which may be due to costs, side effects, inability to understand how to use inhalers or negligence [12]. Indicators of poor asthma control are high consumption of rescue medication (SABA), need for repeated oral steroid treatments, visits to the emergency department, and hospitalizations because of asthma [11].

In Sweden, the overall asthma prevalence is approximately 8% [13]. The present observational study focuses on the prevalence of severe and mild to moderate asthma in Swedish primary care and the characterization of these patients regarding asthma control, management and co-morbidities.

## Methods

### Study design

This observational cohort study linked primary care medical records data to data from national mandatory Swedish health registries.

All asthma patients were identified at 36 primary care centers. Electronic medical records data (e.g. date of birth, sex, diagnoses by ICD-10-CM codes, primary healthcare center contacts, lung function assessments and drug prescriptions) was extracted from 2006 to 2013 using an established software system (Pygargus Customized eXtraction0, Program, CXP™) [14]. Data were also extracted from Swedish national health registries, covering mandatory individual health data on a full population level. Data regarding morbidity and mortality were collected from the National Patient Register, inpatient hospital care (admission and discharge dates, main and secondary diagnoses specified by ICD-10-CM code) and outpatient hospital care (number of contacts, diagnoses), and the Cause of Death Register (date and cause[s] of death), respectively. Data on drug prescriptions from hospital and primary care were collected from the Swedish Prescribed Drug Register. The personal identification number used to identify included patients was replaced with a study ID number prior to further data processing. The Swedish National Board of Health and Welfare performed data linkage. The Department of Medical Sciences, Respiratory Medicine at Uppsala University, Sweden, managed the linked database. The study protocol was approved by the regional ethics committee in Uppsala, Sweden (reference number 2014/446).

### Study population

The population included males and females ≥18 years of age with a record of drug collection for obstructive pulmonary diseases (Anatomical Therapeutic Chemical (ATC) code R03) during 2011–2012, and a physician-diagnosed asthma (ICD-10 code J45-J46) established prior to drug collection. Patients with a diagnosis of polymyalgia rheumatica (ICD-10 code M35.3) or rheumatoid arthritis (ICD-10 code M05) were excluded minimizing the number of patients using oral corticosteroid for other causes than asthma. Index date was defined as first collection of an R03 drug during 2011–2012 and the patients were followed 1 year post index date.

Classification of asthma severity was based on the R03 collection at index date. Severe asthma was classified as collection of high dosage inhaled steroid (ICS, >800 budesonide or equivalent per day) and LTRA and/or LABA collected at the same time or within a 3 months’ period before or after the ICS. Patients who did not fulfil the severe asthma criteria were classified as having mild to moderate asthma.

### Outcomes and variables

Asthma exacerbations were defined as asthma-related hospitalizations (ICD-10 code J45 or J46 as primary diagnosis), hospital emergency visits because of asthma, and/or collection of oral steroids (ATC code H02AB). Exacerbations occurring within a 14 days’ period were calculated as one event. Poor asthma control was defined as either collection of more or equal to 600 doses of SABA, and/or ≥1 exacerbations during the year post index date.

Visits in primary care and outpatient specialist care (to physician or nurse) was defined by a recorded asthma diagnosis in the medical record or registry (ICD-10 code J45 or J46). Mortality was registered post index date to December 31, 2013, i e a follow up duration of 1–2 years.

### Statistical analysis

Statistical analyses were performed using SAS version 9.3 and R version 3.2.3. Baseline characteristics are described as mean (standard deviation, SD) for continuous variables and absolute and relative frequencies for categorical variables. Crude and age adjusted data were calculated. Because of differences in age distribution between groups, the baseline tables were age-standardized using a direct age standardization (i e all observations were weighted relative to the proportion of patient in the specific age group in the total population). Comparisons of baseline characteristics of the age-adjusted values were performed using Chi-square tests. Comparisons of the two asthma severity groups regarding rates of exacerbations and asthma control for the year after index were performed using a Poisson regression, adjusted for age.

## Results

**Fig. 1:**
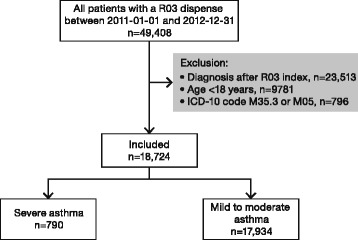
Study flow chart. Out of 49,408 patients with asthma (ICD10 code J45 or J46) who, during 2011 and 2012, collected respiratory drugs (ATC R03) from the pharmacy 18,724 fulfilled the inclusion criteria, i e were above the age of 18 years, had an asthma diagnosis prior to drug collection and did not have polymyalgia rheumatic or rheumatoid arthritis. Of these 18,724 patients, 790 were classified as having severe asthma and 17,934 as having mild to moderate asthma

A total of 18,724 adult patients with asthma were included in this cohort. The mean age was 49.1 years, 62.8% were women, and post-bronchodilator FEV_1_ was 82.6% of predicted value. Severe asthma was identified in 790 patients (4.2%), and 17,934 patients had mild to moderate asthma (Table 1A, Fig. 1). As a control pf consistency it was found that 96% of the patients with severe asthma collected high doses of inhaled steroids also the year after index date. Patients with severe asthma were older at index date (*p* < 0.001) and at asthma diagnosis (*p* < 0.001), and had lower FEV_1_ and FVC than patients with mild to moderate asthma (*p* < 0.001, Table 1A).

**Table 1:** Basal characteristics one year prior to or at index date of (A) all patients with mild to moderate and severe asthma and (B) after exclusion of patients with a COPD diagnosis (J44). Study population and sex are given as number and proportions (%) of patients and other data are given as mean values (standard deviation, SD). *P*-values indicate differences between mild to moderate and severe asthma in each group, respectively

	A			B		
	All patients (*n* = 18,724)			Asthma only (no COPD) (n = 16,703)		
	Mild to moderate	Severe	*P* value	Mild to moderate	Severe	*P* value
Study population, n (%)	17,934 (95.8)	790 (4.2)	<0.001	16,142 (96.6)	561 (3,4)	<0.001
Female, n (%)	11,293 (63.0)	472 (59.7)	0.071	10,157 (62.9)	337 (60.1)	0.169
Age at index	48.8 (19.2)	56.9 (16.9)	<0.001	46.6 (18.6)	52.3 (16.8)	<0.001
Age at death (years)	78.0 (13.2)	81.0 (8.6)	0.042	77.2 (15.1)	81.0 (10.2)	0.165
BMI (kg/m^2^)^a^	27.6 (6.0)	27.9 (5.8)	0.274	27.6 (6.0)	28.0 (5.6)	0.248
FEV_1_% predicted value post bronchodilation^b^	83.6 (21.1)	67.5 (25.9)	<0.001	89.7 (15.9)	80.0 (22.1)	0.003
FVC % predicted value post bronchodilation^b^	94.7 (18.6)	84.3 (21.1)	<0.001	97.7 (16.5)	91.1 (19.8)	0.043
FEV_1_/FVC^b^	0.737 (0.138)	0.640 (0.175)	<0.001	0.777 (0.097)	0.715 (0.140)	0.005
Eosinophils^c^, ×103 cells/μL	0.32 (0.43)	0.40 (0.63)	0.065	0.31 (0.44)	0.37 (0.43)	0.132

^a^BMI was available in 53.0% of patients with mild to moderate asthma and 58.2% with severe asthma

^b^Available data (<10% of the patients in both groups) from lung function measurements performed during the year prior to index date in both groups

^c^Data on blood eosinophilia was available in 16.8% of patients with mild to moderate asthma and 23.8% with severe asthma

### The year prior to index date

During the year prior to index date, 100 exacerbations/100-patient-years were observed for severe asthma and 34/100-patient-years for mild to moderate asthma (Relative Risk, RR [95% confidence interval] 2.59 [2.41–2.79]; *p* < 0.001). Hospitalizations because of asthma were 3.0/100-patient-years for severe asthma and 0.6/100-patient-years for mild to moderate asthma (RR 4.84 [3.09–7.57]; *p* < 0.001). Mean number of oral steroid claims were 97/100-patient-years for severe asthma and 33/100-patients years for mild to moderate asthma (RR 2.57 [2.38–2.77]; p < 0.001). No difference between the groups was found regarding emergency visits at hospital because of asthma exacerbations (RR 0.67 [0.09–4.93]; *p* = 0.694).

In the severe asthma group, one-fifth of the patients had visited secondary care, and one third had visited primary care because of asthma. Visits because of asthma were more frequent in severe asthma than in mild to moderate asthma, while total visits (all causes) to primary care did not differ between the groups (Table 2A).

**Table 2:** Health care utilization one year prior to index date in (A) all patients with mild to moderate and severe asthma and (B) in patients after exclusion of the patients with a concurrent COPD diagnosis. Age adjusted data presented as number and proportion (%) of the patients

	A			B		
	Full study population			Asthma only (no COPD)		
	Mild to moderate (*n* = 17,934)	Severe (*n* = 790)	*P* value	Mild to moderate (*n* = 16,142)	Severe (*n* = 561)	*P*-value
Primary care visits (any reason)						
Visit to the doctor	10,922 (60.9)	464 (58.7)	0.237	9553 (59.2)	312. (55.7)	0.100
Visits to the nurse	8761 (48.9)	376 (47.6)	0.512	7555 (46.8)	248 (44.1)	0.242
Other visits	4880 (27.2)	216 (27.4)	0.968	4065 (25.2)	135 (23.7)	0.458
Primary care visits because of asthma						
Visit to the doctor	3819 (21.3)	245 (31.0)	<0.001	3421 (21.2)	179 (32.0)	<0.001
Visits to the nurse	749 (4.2)	53 (6.7)	0.001	641 (4.0)	36 (6.5)	0.005
Other visits	513 (2.9)	37 (4.7)	0.004	380 (2.4)	23 (4.0)	0.012
Outpatient hospital visits						
Any visit to specialist	3644 (20.3)	296 (37.5)	<0.001	3016 (18.7)	197 (35.1)	<0.001
Visit to a specialist because of asthma	1168 (6.5)	165 (20.9)	<0.001	1068 (6.6)	114 (20.3)	<0.001
Asthma related events						
Exacerbations because of asthma	3182 (17.7)	301 (38.0)	<0.001	2620 (16.2)	196 (35.0)	<0.001
Oral steroids	3111 (17.3)	292 (36.9)	<0.001	2562 (15.9)	190 (33.8)	<0.001
Hospitalizations because of asthma	93 (0.5)	21 (2.6)	<0.001	72 (0.4)	14 (2.5)	<0.001
Emergency department visits because of asthma	22 (0.1)	2 (0.3)	0.620	20 (0.1)	2 (0.4)	0.367

During the year prior to index date, 88.1% in the severe and 65.2% in the mild to moderate asthma group collected any R03 medication (*p* < 0.001, Table 3A). Short-acting beta-agonists (SABA) were collected by half of the patients with severe asthma and for one-third of the patients with mild to moderate disease (*p* < 0.001, Table 3A). Inhaled steroids (mono or fixed combination) were collected by 85.4% with severe asthma and by 55.6% with mild to moderate asthma (p < 0.001, Table 3A). Inhaled steroids were used in combination with LABA in 29.8% of patients with mild to moderate asthma and in 60.4% of those with severe disease while the combination of ICS and leukotriene modifiers (montelukast) was used by 0.6% and 0.9% in mild to moderate and severe disease, respectively. Antihistamines and omalizumab as well as antibiotics alone or in combination with oral steroids were more often collected by patients with severe than for patients with mild to moderate asthma (*p* < 0.001, Table 3A). Patients with severe asthma often collected hypnotics, bisphosphonates, calcium and vitamin D (Table 3A; *p* ≤ 0.002).

**Table 3:** Medication collected during the year prior to index date in (A) all patients with mild to moderate and severe asthma and in (B) in patients after exclusion with a concurrent COPD diagnosis. Age adjusted data presented as number (%)

	A			B		
	Full study population			Asthma only (no COPD)		
	Mild to moderate (*n* = 17,934)	Severe (*n* = 790)	*P* value	Mild to moderate (*n* = 16,142)	Severe (*n* = 561)	*P*-value
Asthma medications	11,695 (65.2)	696 (88.1)	<0.001	10,063 (62.3)	487 (86.8)	<0.001
Short-acting β_2_-agonists (SABA)	5984 (33.4)	394 (49.9)	<0.001	5180 (32.1)	280 (49.9)	<0.001
Inhaled corticosteroids (ICS)	5326 (29.7)	164 (20.8)	<0.001	4779 (29.6)	123 (21.9)	<0.001
Long-acting β_2_-agonists (LABA)	2052 (11.4)	107 (13.5)	0.079	1700 (10.5)	79 (14.0)	0.009
Short acting anticholinergics	345 (1.9)	25 (3.1)	0.020	144 (0.9)	12 (2.1)	0.005
Long acting anticholinergics	814 (4.5)	104 (13.2)	<0.001	160 (1.0)	14 (2.5)	0.001
Fixed ICS/LABA combination	4631 (25.8)	511 (64.6)	<0.001	3723 (23.1)	351 (62.6)	<0.001
Salbutamol and ipratropium^a^	169 (0.9)	25 (3.2)	<0.001	62 (0.4)	11 (2.0)	<0.001
Montelukast^b^	1005 (5.6)	169 (21.4)	<0.001	878 (5.4)	130 (23.2)	<0.001
Methylxanthines	76 (0.4)	14 (1.7)	<0.001	48 (0.3)	4 (0.8)	0.176
Anti-IgE treatment (omalizumab)	0 (0.0)	7 (0.9)	<0.001	0 (0.0)	6 (1.1)	<0.001
Systemic corticosteroids (OCS)	3132 (17.5)	295 (37.4)	<0.001	2581 (16.0)	192 (34.2)	<0.001
N-acetylcysteine	2226 (12.4)	191 (24.2)	<0.001	1521 (9.4)	105 (18.7)	<0.001
Antibiotics and oral steroids^c^	697 (3.9)	124 (15.7)	<0.001			
Cardiovascular medications	6437 (35.9)	336 (42.5)	<0.001	5126 (31.8)	211 (37.6)	0.004
Anti-dyslipidemics	2453 (13.7)	119 (15.0)	0.292	1908 (11.8)	69 (12.3)	0.780
Anti-hypertensives	4478 (25.0)	224 (28.3)	0.035	3471 (21.5)	137 (24.3)	0.110
Beta blockers	2474 (13.8)	122 (15.4)	0.208	1873 (11.6)	76 (13.6)	0.179
Other medications	5152 (28.7)	257 (32.5)	0.023	4025 (24.9)	156 (27.9)	0.135
Antibiotics	3355 (18.7)	209 (26.5)	<0.001	2704 (16.7)	120 (21.3)	0.005
Antihistamines	4437 (24.7)	295 (37.3)	<0.001	4117 (25.5)	224 (40.0%)	<0.001
Nasal corticosteroids	3744 (20.9)	244 (30.9)	<0.001	3458 (21.4)	191 (34.1)	<0.001
Antidepressant	3039 (16.9)	155 (19.6)	0.056	2565 (15.9)	103 (18.4)	0.131
Hypnotics	1989 (11.1)	116 (14.7)	0.002	1592 (9.9)	75 (13.4)	0.008
Antianxiety	2778 (15.5)	134 (16.9)	0.286	2196 (13.6)	78 (13.8)	0.888
Bisphosphonates	343 (1.9)	34 (4.3)	<0.001	231 (1.4)	14 (2.4)	0.060
Calcium D vitamine	63 (0.3)	9 (1.2)	0.001	57 (0.4)	4 (0.8)	0.302

^a^Salbutamol and ipratropium inhaled in a fixed combination

^b^Montelukast was the only available leukotriene modifier on the market

^c^Antibiotics and oral steroids dispensed concomitantly

During the year prior to index date, almost half of the patients in both groups experienced acute upper respiratory tract infections (Table 4A). Chronic bronchitis, nasal polyps, and influenza were more common in the severe asthma group (Table 4A). A concurrent diagnosis of chronic obstructive pulmonary disease (COPD) was found in 21.6% with severe and 10.2% with mild to moderate asthma (*p* < 0.001, Table 4A). Pneumonia was diagnosed in 20.7% of the patients with severe asthma and in 14.4% of the patients with mild to moderate asthma (*p* < 0.001; Table 4A). Heart failure was more common in the severe asthma group (Table 4A).

**Table 4:** Comorbidities during the year prior to index date in (A) all patients with mild to moderate and severe asthma and in (B) patents after exclusion of concurrent COPD diagnosis. Age adjusted data presented as number (%)

	A			B		
	Full study population			Asthma only (no COPD)		
	Mild to moderate (*n* = 17,934)	Severe (*n* = 790)	*P*-value	Mild to moderate (*n* = 16,142)	Severe (*n* = 561)	*P*-value
Respiratory related conditions						
Acute upper respiratory tract infections	8472 (47.2)	378 (47.8)	0.765	7667 (47.5)	271 (48.3)	0.738
Rhinitis, any	3924 (21.9)	173 (21.9)	1.000	3764 (23.3)	133 (23.7)	0.870
Chronic sinusitis	204 (1.1)	14 (1.8)	0.145	176 (1.1)	11 (2.0)	0.085
Nasal polyps	548 (3.1)	42 (5.3)	0.001	493 (3.1)	33 (5.9)	<0.001
Acute lower respiratory infections	4346 (24.2)	204 (25.8)	0.329	3609 (22.4)	131 (23.3)	0.615
Chronic bronchitis	449 (2.5)	32 (4.1)	0.010	256 (1.6)	15 (2.8)	0.067
COPD	1821 (10.2)	171 (21.6)	<0.001	0 (0.0)	0 (0.0)	1.000
Influenza	108 (0.6)	10 (1.2)	0.038	91 (0.6)	6 (1.0)	0.205
Pneumonia	2574 (14.4)	164 (20.7)	<0.001	2011 (12.5)	93 (16.5)	0.005
Other conditions	8625 (48.1)	392 (49.6)	0.421	7143 (44.2)	251 (44.7)	0.852
Diabetes type 1	453 (2.5)	18 (2.3)	0.750	346 (2.1)	13 (2.3)	0.896
Diabetes type 2	1198 (6.7)	54 (6.8)	0.922	890 (5.5)	34 (6.0)	0.643
Arterial hypertension	4450 (24.8)	197 (25.0)	0.971	3435 (21.3)	117 (20.9)	0.850
Ischaemic heart disease	1332 (7.4)	69 (8.7)	0.195	903 (5.6)	35 (6.2)	0.576
Heart failure	760 (4.2)	47 (5.9)	0.026	439 (2.7)	17 (3.0)	0.755
Malignant neoplasm	20 (0.1)	0 (0.0)	0.702	15 (0.1)	0 (0.0)	0.996
Cerebrovascular diseases	565 (3.2)	29 (3.7)	0.476	394 (2.4)	15 (2.7)	0.832
Anxiety and depression disorder	3682 (20.5)	161 (20.4)	0.954	3198 (19.8)	110 (19.6)	0.948
Osteoporosis	382 (2.1)	22 (2.8)	0.265	244 (1.5)	10 (1.7)	0.734
Inflammatory bowel disease	607 (3.4)	22 (2.8)	0.415	526 (3.3)	14 (2.4)	0.377

### The year after index date

**Fig. 2:**
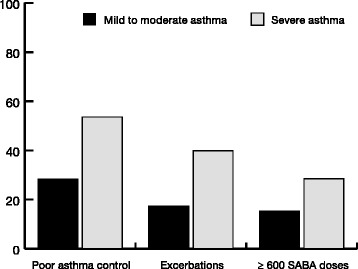
Poor asthma conttrol assessed by acute exacebations and the collection of more than 600 doses of short-acting beta-2 agonists the year after index date

During 1 year after index date, poor asthma control was observed for 28.2% of the patients with mild to moderate asthma and for 53.6% of the patients with severe asthma (<0.001, Fig. 2). In the mild to moderate and severe group, 15.2% and 28.6% of the patients, respectively (*p* < 0.001), collected more than 600 doses of SABA (Fig. 2).

Inhaled steroids, in any form, was used by 78.1% of the patients with mild to moderate asthma and 95.9% of those with severe asthma (*p* < 0.001 between the groups). Health care utilization and exacerbations were similar the years before and after index date.

Overall, 640 (3.6%) patients in the mild to moderate group and 42 (5.3%) patients in the severe asthma group died during the observation period post-index. The most common causes of death were cardiovascular disease (33% vs 43%), neoplasms (27% vs 17%) and respiratory diseases (18% vs 14%) in mild to moderate asthma and severe asthma, respectively. COPD was the most common respiratory cause of death (81% and 83%, respectively). Nine patients in the mild to moderate group (8% of the respiratory deaths) died from asthma, whereas no asthma deaths were observed in the severe group.

### Sensitivity analyses

#### Patients with no concurrent COPD diagnosis

Patients with asthma only (i e no concurrent COPD diagnosis, *n* = 16,703) were younger and had greater FEV_1_ and FVC than the full population (Table 1B). The difference of health care utilization remained similar between mild to moderate and severe asthma (Table 2 B) whereas asthma medication (except for xanthines) was more frequently used in severe asthma (Table 3B). For patients with no concomitant COPD diagnosis long-acting anticholinergics were collected for 1.6% in the mild to moderate group and for 5.2% in the severe group. Use of bisphosphonates and vitamin D did not differ between severe and mild to moderate asthma (Table 3B). In the asthma only group, the risk of pneumonia remained greater in severe asthma whereas the prevalence of chronic bronchitis and heart failure did not differ between severe and mild to moderate asthma (Table 4B). Asthma control was better for patients with mild to moderate than for patients with severe asthma (Table 5A).

**Table 5:** Patients with mild to moderate and severe asthma excluding patients with a concurrent COPD diagnosis (asthma only) and asthma patient who also have a COPD diagnosis (“Both asthma and COPD”). Age adjusted data the year after index date. Mean cumulative event indicate is presented as events/100-patient-years. “Mild to moderate A versus B” and “Severe A versus B” indicate comparisons between patients with asthma only and “Both asthma and COPD” with mild to moderate and severe asthma, respectively

	A Asthma only (no COPD)			B Both asthma and COPD			C	
							Mild to moderate A vs B	Severe A vs B
	Mild to moderate	Severe	*P* value	Mild to moderate	Severe	*P* value	*P* value	*P* value
Study population, n (%)	16,007 (96.6)	558 (3.4)		1680 (88.3)	222 (11.7)			
Poor asthma control, n (%)	4250 (26.5)	283 (50.8)	<0.001	743 (44.2)	130 (58.6)	<0.001	<0.001	0.018
≥600 SABA, n (%)	2249 (14.0)	154 (27.7)	<0.001	442 (26.3)	67 (30.1)	0.253	<0.001	0.324
Exacerbation, n (%) Mean cumulative event	2595 (16.2)	203 (36.3)	<0.001	483 (28.8)	101 (45.4)	<0.001		
	29.09	87.46	<0.001	76.31	152.25	<0.001	<0.001	0.001
Oral steroids, n (%) Mean cumulative event	2566 (16.0)	203 (36.3)	<0.001	481 (28.7)	100 (44.9)	<0.001		
	28.71	84.41	<0.001	75.06	150	<0.001	<0.001	0.006
Hospitalization, n (%) Mean cumulative event	42 (0.3)	7 (1.2)	<0.001	16 (0.9)	4 (2.0)	0.414		
	0,3	2.33	<0.001	1.13	2.25	0.17	<0.001	0.035
ED visit, n (%) Mean cumulative event	12 (0.1)	5 (0.9)	<0.001	1 (0.1)	0 (0.0)	1.000		
	0.07	0.72	<0.001	0.12	0	0.996	0.529	0.478
Asthma medications, n (%)	12,247 (76.5)	525 (94.2)	<0.001	1582 (94.2)	217 (97.9)	0.040	<0.001	<0.001
Inhaled corticosteroids (ICS), n (%)	6069 (37.9)	139 (24.9)	<0.001	538 (32.0)	37 (16.7)	<0.001	<0.001	<0.001
Long-acting ß2-agonists (LABA), n (%)	1992 (12.4)	97 (17.4)	0.001	352 (21.0)	30 (13.6)	0.012	<0.001	<0.001
Fixed ICS/LABA combination, n (%)	4567 (28.5)	409 (73.3)	<0.001	950 (56.6)	188 (84.7)	<0.001	<0.001	<0.001
Long-acting anticholinergics, n (%)	260 (1.6)	29 (5.2)	<0.001	669 (39.8)	120 (54.1)	<0.001	<0.001	<0.001
Leukotriene modifiers (LTRA), n (%)	1091 (6.8)	171 (30.7)	<0.001	128 (7.6)	35 (15.6)	<0.001	<0.001	<0.001
Methylxanthines, n (%)	43 (0.3)	6 (1.0)	0.002	27 (1.6)	9 (4.0)	0.024	0.050	0.05
Short-acting ß2-agonists (SABA), n (%)	6635 (41.5)	326 (58.5)	<0.001	828 (49.3)	113 (50.9)	0.703	<0.001	<0.001
Systemic corticosteroids (OCS), n (%)	2698 (16.9)	209 (37.5)	<0.001	493 (29.4)	102 (45.8)	<0.001	<0.001	<0.001
Omalizumab, n (%)	1 (0.0)	5 (0.9)	<0.001	0 (0.0)	0 (0.0)	1.000	1.000	1
N-acetylcysteine, n (%)	1600 (10.0)	114 (20.4)	<0.001	626 (37.3)	112 (50.5)	<0.001	<0.001	<0.001
Primary care visits because of asthma, physician, n (%)	3491 (21.8)	153 (27.4)	0.002	291 (17.3)	44 (20.0)	0.410	0.001	0.009
Primary care visits because of asthma, nurse, n (%)	799 (5.0)	35 (6.2)	0.207	93 (5.5)	9 (4.2)	0.446	0.604	1
Primary care visits because of asthma, other, n (%)	418 (2.6)	23 (4.2)	0.041	98 (5.8)	19 (8.6)	0.150	<0.001	0.171
Specialist outpatient visits because of asthma, n (%)	980 (6.1)	118 (21.1)	<0.001	87 (5.2)	21 (9.7)	0.015	<0.001	0.033

#### Patients with both asthma and COPD diagnosis

For patients with both asthma and COPD diagnosis (*n* = 1902), exacerbation rate was greater for those with severe than for those with mild to moderate asthma, while hospitalization, emergency department visits and asthma control assessed as the use of SABA did not differ (Table 5B). Visits to secondary care were more frequent in severe than in mild to moderate asthma but visits to primary care did not differ (Table 5B). The use of asthma drugs was greater in the severe group, except for SABA (Table 5). For patients with both asthma and COPD diagnoses, long-acting anticholinergics was collected by 39.8% of those with mild to moderate asthma and 54.1% of those with severe asthma. In patients with both diagnoses 31% in the mild to moderate group and 37% in the severe group had pneumonia the year prior to index date (*p* = 0.084 between groups).

#### Asthma only patients vs patients with both asthma and COPD diagnoses

For asthma patients with a concurrent COPD diagnosis, exacerbations rate was greater than it was for the group with asthma only irrespective of asthma severity, while asthma control assessed as use of SABA was worse in the mild to moderate group only (Table 5C).

The use of asthma drugs (except for ICS as monotherapy) was more frequent in the group with both asthma and COPD diagnosis than in the asthma only group irrespective of asthma severity (Table 5C). Visits to primary care physician because of asthma and visits to secondary care were more frequent in the asthma only group than in patients with both asthma and COPD diagnosis irrespective of asthma severity, while visits to a nurse or other visits (e g physiotherapists) did not differ (Table 5C).

## Discussion

In this observational cohort study, covering 18,724 asthma patients, the prevalence of severe asthma was 4.2%, which is somewhat lower than the commonly reported prevalence of 5–-10% [15, 16]. For severe asthma, the exacerbation rate was twice as high and poor asthma control twice as common as for mild to moderate asthma. In severe asthma, only one-fifth of the patients had visited secondary care and one third had a visited primary care because of asthma.

The prevalence of severe asthma was similar to a previous study, where 3.6% of patients with asthma had severe refractory asthma [17], but lower than in another study which reported a prevalence of 8.1% [11]. In that study, patients were included on a population basis, while patients were included from primary care records in our study. This may result in an underestimation of patients with severe asthma, as patients who are managed in secondary care only will not be included. In the present study, all patients above the age of 18 years were included, whereas patients above the age of 44 years were excluded in the Danish study [11]. As we did not exclude patients with a concurrent COPD diagnosis, the mean age was rather high at index date. Inclusion of older patients may have implied a “dilution” effect because of a reluctance to classify severe respiratory disease as asthma in elderly patients.

Poor asthma control (exacerbations, need for oral steroids, emergency department visits, collection of more than 600 doses of SABA during the last year) was observed for 53% of the patients with severe asthma, twice as many as in the mild to moderate asthma group. In a Danish study, with identical definition of asthma control as in our study, poor asthma control was observed for 36% of patients with severe asthma [11]. In that study, only patients below 44 years of age were included and the prevalence of severe asthma was twice as high as in the present study. These findings indicate that the older asthma population in the present study, despite similar definition of severe asthma in the two studies, had more severe disease than the younger population in the Danish study.

Exacerbation rate was twice as high, visits to primary care because of asthma were 50% greater and visits to secondary care because of asthma were three times more frequent in the severe asthma group as in the mild to moderate group. In addition, the collection of asthma medication was greater in the severe asthma group than they were for the mild to moderate group. All these differences between mild to moderate and severe asthma remained also after excluding patients with COPD clearly indicating that the high exacerbation rate, the increased utilization of health care resources and increased need for medication were mainly driven by asthma severity and not by co-existing COPD. In our study, the prevalence of COPD in the group with severe asthma was 21.6%, which is similar to what has been reported by others [18].

Co-existing COPD increased the risk of exacerbations, the need for health care utilization and need for medication irrespective of asthma severity. This is in accordance with previous studies where the risk of exacerbations and hospitalization were greater in patients with features of both asthma and COPD than in patients with COPD or severe asthma [18–20]. The diagnoses were based on the physicians’ judgements, indicating that patients with both diagnoses exhibited features of both asthma and COPD. Although there is likely a substantial agreement between having both an asthma and COPD diagnoses and having asthma-COPD overlap (ACO) according to GINA guidelines [2], we cannot, from the present results, assert that having both diagnosis is equal to the ACO concept. Hence, co-existing COPD increased the disease burden even more as patients with both asthma and COPD, compared with asthma only, had an increased disease burden in all these aspects unrelated to asthma severity.

As expected the asthma control was better in the mild to moderate group than in the severe group. Half of the patients with severe asthma and one of four patients with mild to moderate asthma had poor asthma control. This is in agreement with previous studies in which approximately half of patients reported daytime symptoms or were regarded as having uncontrolled asthma as assessed by the Asthma Control Questionnaire [3, 4]. Our data confirm previous results showing that a substantial number of patients with mild or moderate asthma have an uncontrolled disease.

Surprisingly, we found 50% greater occurrence of pneumonia in the group with severe asthma than in the mild to moderate asthma group. Treatment with ICS increases the risk of pneumonia in COPD [21] although there are intra-class differences between different steroids [22, 23]. O’Byrne et al. did not find an increased risk for pneumonia in asthma patients treated with ICS [24], whereas other results indicate an association between treatment with inhaled steroids and pneumonia also in asthma [25]. The difference in pneumonia risk was increased in severe asthma vs mild to moderate asthma after exclusion of patients with a concurrent COPD diagnosis. The risk of pneumonia was also greater in the groups with a concurrent COPD diagnosis than in patients without a COPD diagnosis. These findings suggest that asthma severity and COPD by itself are associated with increased risk of pneumonia.

According to international and Swedish recommendations, severe asthma should be managed by specialists/physicians with extensive experience of asthma management [2, 8]. In the present study, only one out of five patients with severe asthma visited a specialist in secondary care in the year before and after index date, and the frequency was independent of a concurrent COPD diagnosis. The attendance rate to doctors in primary care was also low. Less than 60% of the asthma patients visited primary care for any reason and close to a third of the patients visited a primary care doctor because of asthma, irrespective of disease severity. This gives reasons to believe that many of these patients are not regularly visiting a doctor or a nurse but are managed by prolongation of asthma drug prescription through telephone contacts. Such a situation is unsatisfactory, particularly for patients with severe asthma.

In the present study, somewhat less than half of the patients with mild to moderate asthma and more than 10% of the patients with severe asthma did not collect inhaled steroids the year prior to index date. The reason for this is unclear. Factors such as treatment failure due to incorrect diagnosis or poor steroid response may contribute. As it could be assumed that the majority of physicians prescribe inhaled steroids to patients with severe asthma, a likely explanation is poor adherence to prescribed treatment. We do not know to what extent the patients were adherent to treatment but we do know that approximately half of the patients with mild to moderate asthma and about 85% of the patients with severe asthma collected inhaled steroids from the pharmacy. The mean age in our study was approximately 50 years and it has been shown that adherence to treatment increases with age [26, 27] indicating that the adherence to therapy may have been high among those who actually did collect their medication. We also conclude that 5 to 15% of the patients with severe asthma did not collect inhaled steroids which most likely contributes to the increased costs as it is well known that poor adherence to asthma treatment is associated with increased health care costs [12]. Our data demonstrate that long-acting anticholinergics are primarily used in asthma patients who also have a COPD diagnosis and not frequently as an add on treatment in asthma patients with uncontrolled asthma. Our results also demonstrate that leukotriene modifiers (montelukast being the only drug registered in Sweden) in addition to inhaled steroids is not frequently used in the treatment of asthma in Sweden, neither in mild/moderate nor in severe asthma.

The present study is based on observational registry and medical records data implying a design that may have its limitations. Data retrieval is limited to the variables registered in the databases and medication use is based on prescription collections, which do not necessary reflect how patients actually use their medications. Furthermore, exacerbation rate may have been underestimated, as it was based on acute visits to secondary care and on collection of oral steroids and acute exacerbation managed at visits to the primary care centers were not recorded. The results validate the classification of severe asthma, by showing five times more hospitalizations in the severe asthma population compared with the mild to moderate group, supporting that this really is a severe asthma population. This is corroborated by the finding that 96% of the patients with severe asthma also collected high doses of inhaled steroids from a Swedish Pharmacies the year after index date. Important strengths of the study is the primary care setting used to identify the asthma patients, and the lack of restrictions in, for example, age, socio-economic status or healthcare insurance. This non-biased data extraction from primary care electronic medical records linked with mandatory national healthcare registers with high coverage and quality provides solid and unique data.

## Conclusion

In conclusion, the prevalence of severe asthma in this Swedish primary care population was 4.2%. Asthma severity increased the disease burden, which was further increased in patients with a concurrent COPD diagnosis. Patients with severe asthma had few regular contacts with both primary and specialist care, and more than half of them experienced poor asthma control.

## Abbreviations

ATC: Anatomical therapeutic classification system code
CI: Confidence interval
COPD: Chronic obstructive pulmonary disease
FEV_1_: Forced expiratory volume in one second
FVC: Forced vital capacity
ICD-10-CM: International Classification of Diseases 10 Clinical Modification
ICS: Inhaled corticosteroid
LABA: Long-acting beta-agonists
LTRA: Leukotriene receptor antagonist
RR: Relative risk
SABA: Short-acting beta-agonists
SD: Standard deviation
